# Novel Microsatellite Markers of *Meretrix petechialis* and Cross-species Amplification in Related Taxa (Bivalvia: Veneroida)

**DOI:** 10.3390/ijms131215942

**Published:** 2012-11-28

**Authors:** Jung-Ha Kang, Byeng-Hak Kim, Jung-Youn Park, Jung-Mi Lee, Ji-Eun Jeong, Jun-Sang Lee, Hyun-Sook Ko, Yong-Seok Lee

**Affiliations:** 1 Biotechnology Research Division, NFRDI, Busan 619-705, Korea; E-Mails: kjh0124@nfrdi.go.kr (J.-H.K.); jypark@nfrdi.go.kr (J.-Y.P.); 2 Southwest Sea Fisheries Research Institute, NFRDI, Yeosu 556-823, Korea; E-Mail: bhkim@nfrdi.go.kr; 3 Gyeongsangnam-do Fisheries Resources Research Institute, Tongyoung 650-974, Korea; E-Mail: leebeauty62@korea.kr; 4 Department of Parasitology, College of Medicine and UHRC, Inje University, Busan 614-735, Korea; E-Mail: jje2010@inje.ac.kr; 5 Institute of Environmental Research, Kangwon National University, Chunchon 200-701, Korea; E-Mail: sljun@kanwon.ac.kr; 6 Department of Biological Science, Silla University, Busan 617-736, Korea; E-Mail: hsko@sila.ac.kr

**Keywords:** *Meretrix*, microsatellite, cross-species, Veneroida

## Abstract

The Asian hard clam, *Meretrix petechialis*, is an economically important bivalve, but its catch and population sizes are decreasing rapidly, owing to many factors, including large-scale reclamation of its natural habitat on the western coast of the Korean peninsula. Attempts to restore the resources and production of this species require genetic structure and diversity information. In this study, we developed 15 microsatellite markers from a partial genomic library enriched in GT repeats. Nine of these markers were polymorphic, with an average allele number of six, and six were monomorphic in 95 tested individuals. No linkage disequilibrium was found between any pair of loci (*p* > 0.05), and deviations from the Hardy–Weinberg equilibrium (HWE) test showing excess of heterozygotes was observed in only one of nine loci. In addition, no null alleles or genetic differentiation between two tested populations were detected. A cross-species amplification in 12 species of four families resulted in two *M. petechialis*-specific loci and three possible universal markers. This information will be useful in the future development of high-quality artificial seedlings and sustainable resource management.

## 1. Introduction

The genus *Meretrix*, commonly known as Asian hard clams, is distributed in the West Pacific, Asia, and the Indian Ocean [1] and is an important commercial bivalve in East and Southeast Asia and East Africa [2]. These clams inhabit tidal flats, estuaries and sandy beaches, which are well developed on the southern and western coasts of the Korean Peninsula. Most of the members of the genus are important fishery resources.

Among the nine recognized species, two very closely related species, *M. lusoria* and *M. petechialis*, occur naturally along the west coast of the Korean Peninsula and China [3] and are economically very important. *Meretrix lusoria* inhabits the southern coast of Korea, and *M. petechialis* occupies the western coasts, with the border between them located along the southwestern coast of Korea from Gangjin Bay to Baeksu [4].

As with other clams, natural populations of *M. petechialis* have decreased drastically on the Korean Peninsula because of ocean pollution and reclamation. The annual production of *M. petechialis* in Korea was 11,705 M/T in 1971, but only 1454 M/T in 2008. Among the many factors affecting the decrease of *M. petechialis* in Korea, the reclamation of the Saemangeum area, with a 33 km long dike, has had a great effect, as this area is the most productive site of *M. petechialis* in the country [5].

In response to the decrease in natural habitat and production of *M. petechialis,* aquaculture production and seed release to restore natural resources have become necessary. The aquaculture of this species has been long attempted, and complete culturing, including reproductive control, captive spawning, hatching, and larval and juvenile rearing, has only recently become possible on a small scale.

Future production of high-quality seedlings for aquaculture and release depends on information regarding the genetic structure and diversity of the species, which can be provided by using reliable genetic markers. However, despite their economic importance, few phylogenetic and population genetic studies of the genus *Meretrix* have been conducted.

Mitochondrial DNA (mtDNA) is the most commonly used target DNA for taxonomic and phylogenetic purposes because of its simple maternal inheritance, absence of recombination and high substitution rate [6]. However, a very high level of sex-associated mtDNA heteroplasmy and unusual doubly uniparental inheritance (DUI) have been reported from different bivalve families, including the Veneridae [7,8]. Thus, special care in the taxonomy of bivalves is required.

In contrast to mtDNA markers typically used for species identification, co-dominant genetic markers, such as microsatellite (MS) markers, are useful for genetic structure analysis, family identification and parentage assignment. The combination of microsatellite markers and genetic improvements in breeding has considerably improved shellfish aquaculture, including oysters, mussels and abalones [9–12]. However, little information is available on the genetic diversity of the genus *Meretrix* based on microsatellite markers, because these useful markers have only recently been applied to the genus.

Microsatellites, also known as single sequence repeats (SSRs), are highly useful because of their abundance, even distribution of short lengths and high polymorphism. Although high-throughput sequencing, called next-generation sequencing (NGS), techniques have been applied to recent MS marker development [13,14], the development of effective MS markers is tedious, labor-intensive and expensive, involving the screening of genomic libraries using repetitive probes and sequencing of positive clones [15]. One way to solve this problem is to apply MS markers developed for a particular species to closely related species, which has been proven useful in fishes [16,17]. In this study, we developed microsatellite markers for *M. petechialis* and tested their transferability to other clams belonging to the order Veneroida.

## 2. Results

### 2.1. Microsatellite Marker Isolation and Polymorphism Analyses

In total, 1,700 positive colonies were obtained from the transformation of the hard clam (GT)*_n_*-enriched genomic DNA library. Of these, 1000 clones were randomly picked and sequenced. Primers were designed and tested for 160 loci that contained a minimum of eight repeat units, and only 50 primer sets produced strongly amplified PCR products. Of those, 35 gave either inconsistent or noise peaks, despite adjusting the dNTP concentration and PCR annealing temperature. The remaining 15 were considered successful genetic markers. Table 1 lists the variability information and GenBank accession numbers of the 15 microsatellite markers. Among these, nine showing polymorphisms in the amplified microsatellite markers were used for further genetic study of two hard clam populations.

### 2.2. Genetic Variability of *Meretrix petechialis* Populations

Table 2 summarizes the genetic characterization indices estimated for the two hard clam populations. The mean allelic richness per locus ranged from 2.0 to 16.3 in the two populations. The average number of alleles in all populations was 6.0. No linkage disequilibrium was found between any pair of loci (*p* > 0.05), indicating that the markers were independent. The Hardy-Weinberg equilibrium (HWE) test, indicating the deviation from expected heterozygosity, showed significant deviation after sequential Bonferroni correction (*p* < 0.01) in the Mp07-nfrdi locus with excess heterozygosity in two populations. No null alleles and no genetic differentiation between GC and MA populations were detected by *F*_ST_ (=0.013, *p* < 0.01) using all nine microsatellite markers.

### 2.3. Cross-Species Amplification

Cross-species amplification of 15 loci was conducted in 12 related species belonging to four families in the order Veneroida (Bivalvia: Heteroconchia). The 12 species were representative of shellfish. As shown in Table 3, two MS markers, Mp04-nfrdi and Mp09-nfrdi, produced PCR products from only *M. petechialis*. No PCR product was obtained from the remaining 11 tested species. In contrast, the marker Mp06-nfrdi produced PCR products from 11 of the 12 tested species, followed by the Mp02-nfrdi and Mp08-nfrdi, which produced PCR products from 10 of the 12 species. These markers were polymorphic in the 5, 6, and 7 species, respectively. The transferability of 15 loci to the 12 related species ranged from 33.3% to 66.7%, with the ratio of polymorphic sites being 6.7%–46.7%. However, some of the markers that were monomorphic in *M. petechialis* were polymorphic in other species, and *vice versa*.

## 3. Discussion

In total, 15 microsatellite markers were obtained from a partial genomic library of *M. petechialis* enriched in GT repeats. Nine were polymorphic, with an average allele number of six, and six were monomorphic in 95 tested individuals. Deviation from HWE equilibrium, including significant deviation in the Mp07-nfrdi locus, indicated excess heterozygosity in two populations. Deviations from HWE have been observed in several marine commercial fish [18] and mollusks [19–22]. Deviation from HWE may be due to one or a combination of factors, including the substructure of samples due to the pooling of samples from many sites, inbreeding or the presence of null alleles [23]. The presence of null alleles produce an excess of homozygotes, and heterozygote deficiency observed in several bivalves, including the geoduck clam, European flat oyster [24] and pink mucket [25], is thought to be due to the presence of null alleles [26]. We did not find any null alleles among the 15 loci tested in *M. petechialis*, which may be one reason for the excess of heterozygosity. This is a major limitation in cross-species transfer of microsatellite markers [27].

The other possible reason of the excess of heterozygosity can be a genetic bottleneck caused by a recent decrease of the population due to ocean pollution and reclamation, such as the reclamation of the Saemangeum area, the most productive site of *M. petechialis* in the country [5]. A population that suffered a recent bottleneck can show an excess of heterozygosity, because alleles are generally lost faster than heterozygosity during a bottleneck [28,29].

Type-specific microsatellite markers, such as Mp04-nfrdi and Mp09-nfrdi, can be useful for species identification, especially at the early larval stage when morphological distinction between these two species is impossible. Torii *et al.*[30] reported that the two species could be differentiated with 98.89% success by modified discrimination scores using five characteristics. They also showed that the matching scores of nucleotide sequences between the two species were 93% and 97% in the mitochondrial *COI* gene and *ITS*-*1* gene, respectively. However, these methods can be applied only to individuals of sufficient size, require time-consuming sequencing procedures and cannot be used on mixed larval samples to detect the presence of specific species. Restriction analysis of specific PCR products can be used to produce specific banding patterns, which can be used for certain purposes, such as the identification of the *M. petechialis*-*M. lusoria* hybrid [31]; however, these techniques cannot be used in mixed samples. Species-specific microsatellite markers have been used to identify specific oyster species in mixtures of oyster larvae [32]. As with other aquatic animals, microsatellite markers have been used to determine parentage in the genus *Meretrix*, either alone [33] or in combination with other genetic markers [34]. The possible use of the two species-specific loci for species identification was further confirmed by a BLAST search of primer sequences, which showed no match to known DNA sequences.

Nuclear microsatellite markers are the most popular genetic markers in population genetic studies, but they need to be developed and optimized for each species, which is time-consuming and somewhat expensive. Thus, markers that can be readily transferred to related species are desirable. We tested the transferability of 15 microsatellite makers developed in *M. petechialis* against 12 species in four families of the order Veneroida. As shown in Table 3, the overall cross-species transferability of the 15 makers to the 12 tested species ranged from 33.3% to 66.7%, with polymorphic loci ranging from 13.3% to 46.7%. Among the 15 markers, three loci, Mp02-nfrdi, Mp06-nfrdi and Mp08-nfrdi, showed an amplification success rate of 90.9%, with a polymorphism ratio of 45.5%–63.6%, suggesting that these markers can be used as universal markers in related species. In the cross species PCR, some of the products in other species were far outside the range of those observed in *M. petechialis*. For example, MP05 has a size range of 78–92 in 95 specimens of *M. petechialis,* but a range of 82–332 in only two specimens of *Cyclina sinensis*. Therefore, further cloning and sequence analysis of these PCR products might be necessary for the confirmation of these PCR products as real alleles. The limited information available regarding cross-species transfer of microsatellite markers developed in bivalves shows the transferability between species within genera to be 93.3% (14 of 15) in *Crassostrea*[35] and 86.7% (13 of 15) in *Lampsilis*[25].

**Figure 1:**
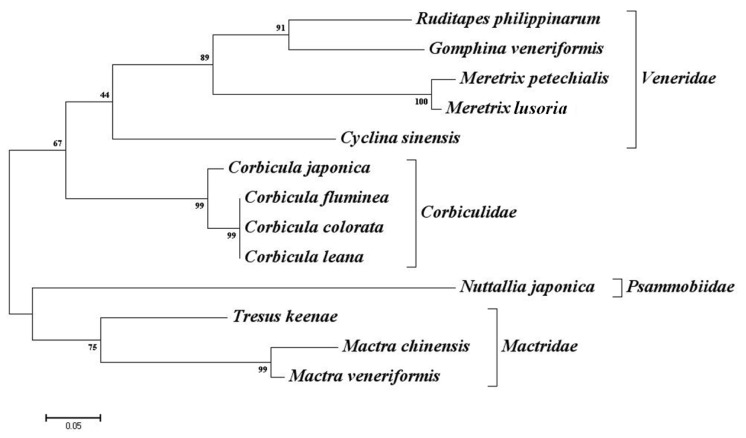
Neighber-Joining tree based on the 16S rRNA sequences. The 16S rRNA sequence for *Corbicula colorata* (JX399588), *Corbicula leana* (JX399587), *Tresus keenae* (JX399585) and *Gomphin veneriformis* (JX399586) was determined in this study. The Genbank accession numbers for rest of the samples are *Ruditapes philippinarum* (JN969951.1), *Meretrix petechialis* (NC012767.1), *Meretrix lusoria* (JN969940.1), *Cyclina sinensis* (DQ356379.1), *Corbicula japonica* (AB304507.1), *Corbicula fluminea* (DQ280039.1), *Nuttallia japonica* (AB476426.1), *Mactra chinensis* (JN674598.1). Numbers near the node indicate boostrap support.

As Barbará *et al.*[27] suggested, the evolutionary distance between the source of microsatellite markers and the target species is the most important factor affecting the success of cross-species amplification. This was confirmed in our study, as shown in Figure 1. Except for the Psammobiidae with one member, transferability was related to the evolutionary distance deduced from the sequence of 16S rRNA, averaging 56.6%, 44.4% and 40% for Veneridae, Mactridae and Corbiculidae, respectively. One exceptional case in our study was the low transferability to *M. lusoria*, the most related species*,* which showed successful amplification at only seven loci. One possible reason could be the genetic homogeneity of the individuals used for the test, but no information regarding the genetic diversity of this species in the Korean Peninsula is available. In an analysis of 611 cross-species microsatellite marker transfers, Barbará *et al.*[27] reported that the percentage of amplified markers among families within the order ranged from 28% to 33%. The transferability of the microsatellite markers developed in our study has a comparably high transferability, and some, such as those listed above, could be used as universal markers in the order Veneroida.

## 4. Experimental Section

### 4.1. Sample Collection

A total of 95 wild hard-clam samples were collected from two locations on the west coast of the Korean Peninsula, Gochang (GC, *N* = 35) and Muan (MA, *N* = 60). Tissue samples from a leg were preserved in 100% ethanol at the sampling site and then transported to the laboratory for DNA extraction. Total DNA was isolated from each sample using a MagExtractor MFX-6100 automated DNA extraction system (Toyobo, Osaka, Japan). The extracted genomic DNA was quantified using a Nanodrop ND-1000 spectrophotometer (Thermo Fisher Scientific, Barrington, IL, USA) and stored at −20 °C until microsatellite genotyping analysis. For the cross-species transferability test, DNA was extracted by the same method from ethanol-fixed tissues of 12 related species belonging to four families of the order Veneroida that had been stored at the National Fisheries Research and Development Institute, Busan.

### 4.2. Library Construction and Sequencing

A partial genomic library enriched in GT repeats was constructed using the procedures described by Hamilton *et al.*[15]. Genomic DNA was isolated from one individual using the TNES-urea buffer method [36]. Extracted DNA was digested with a restriction enzyme mixture containing *Alu*I, *Rsa*I, *Nhe*I and *Hha*I (New England Biolabs, Beverly, MA, USA). Following agarose gel electrophoresis, DNA fragments of 200–800 base pairs (bp) were isolated from the gel and ligated to an adapter SNX/SNX rev linker [15]. The linker-ligated DNA was amplified using SNX as a linker-specific primer for polymerase chain reaction (PCR). For enrichment, the DNA was denatured, hybridized to biotin-labeled repeat sequence (GT)_10_ probes and then attached to streptavidin-coated magnetic beads (Promega, Madison, WI, USA). The enriched fragments were digested with the enzyme *Nhe*I, and ligated into the *Xba*I-digested pUC18 vector (Pharmacia, Piscataway, NJ, USA), followed by transformation into *Escherichia coli* DH5α-competent cells. The positive colonies were screened for the presence of a repeat insert using PCR with universal M13 primers and non-biotin-labeled dinucleotide primers. PCR products were examined on 2% agarose gels, and inserts producing two or more bands were considered to contain a microsatellite locus. Positive clones were cultured, and plasmids from insert-containing colonies were recovered using the QIAprep Spin Miniprep Kit (Qiagen) and sequenced using the BigDye Terminator Cycle Sequencing Ready Reaction Kit (ver. 3.1; Applied Biosystems, Foster City, CA, USA) and an automated sequencer (ABI Prism 310 Genetic Analyzer; Applied Biosystems).

### 4.3. Primer Design and Genotyping

Primers were designed based on sequences flanking the microsatellite motifs using the PRIMER 3 software package. Newly designed PCR primer pairs were tested to optimize annealing temperatures: a gradient PCR at 50–60 °C annealing temperature range was performed on a set of samples from eight individuals. PCR amplification was performed in a 10 μL reaction mixture containing 0.25 U *Extaq* DNA polymerase (TaKaRa Biomedical Inc., Shiga, Japan), 1 × PCR buffer, 0.2 mM dNTP mix, 10 pmol each primer (forward primer of each pair was 5′-end-labeled with 6-FAM, NED and HEX dyes; PE Applied Biosystems) and 100 ng template DNA, using a PTC 200 DNA Engine (MJ Research, Waltham, MA, USA). PCR conditions were as follows: 11 min at 95 °C, followed by 35 cycles of 1 min at 94 °C, 1 min at the annealing temperature listed in Table 2, and 1 min at 72 °C, with a final extension of 5 min at 72 °C. Microsatellite polymorphisms were screened using an ABI PRISM 3130 XL automated DNA sequencer (Applied Biosystems), and alleles were designated according to PCR product size, relative to a molecular size marker (GENESCAN 400 HD [ROX]; PE Applied Biosystems). Fluorescent DNA fragments were analyzed using GENESCAN.

### 4.4. Statistical Analysis

The number of alleles per locus, allele frequency and heterozygosity were calculated using CERVUS 3.03 [37]. Tests for population-wide linkage disequilibrium between pairs of loci and deviations from HWE were estimated using GENEPOP ver.4.0 [38], and the adjusted *P*-values for both analyses were obtained using a sequential Bonferroni test for multiple comparisons [39]. MICRO-CHECKER 2.2.3 [40] was used to test for the presence of null alleles. Allelic richness (*A*_R_) as a standardized measure of the number of alleles per locus, independent of the sample size, was calculated using FSTAT version 2.9.3 [41].

## 5. Conclusions

To our knowledge, this is the first report on the development of microsatellite markers for *M. petechialis*. As the aquaculture of this species is at an early stage, this study will be helpful in the future genetic improvement of culture stocks and in managing the natural resource of this economically valuable, but threatened, species. In addition, some of the microsatellite markers could be used as universal genetic markers for other species.

## Figures and Tables

**Table 1:** Characteristics of 15 microsatellite loci isolated from *Meretrix petechiails*.

Primer name	Core sequence		Primer (5′→3′)	Annealing temp.	Amplicon size	No. Allele	*H*o	*H*e	GenBank accession No.
Mp01-nfrdi	(AC)_34_	**F** **R**	GACCGCGATCGTATACAAGTCCC TCCGTGCATGTGTGCCTATATCC	48	179–199	4	0.372	0.306	JX017338
Mp02-nfrdi	(AC)_14_	**F** **R**	CATGGGAAGCAGGCGGTTTGTTG ACATACGTGTGCGCATGCGTGTG	52	102–164	9	0.327	0.287	JX017339
Mp03-nfrdi	(AC)_29_	**F** **R**	TCGTATACAAGTCCCGGTCCTGG TCCGTGCATGTGTGCCTATATCC	50	149–155	2	0.118	0.111	JX017340
Mp04-nfrdi	(AC)_24_	**F** **R**	GGATTCCAGTTTAGCCCTCTC TATACACAGCGCAAGGTGAAC	52	246–286	13	0.244	0.218	JX017341
Mp05-nfrdi	(AC)_28_	**F** **R**	CCATATTTGACAGCAGTTTCGTC CAAAGTATCGCCTGAACCTGAC	50	78–92	6	0.369	0.34	JX017342
Mp06-nfrdi	(AC)_11_	**F** **R**	ACAGGACCTGATCGTGAACAC CAGGCCGAGTGCAGAAGTGGA	50	168–196	2	0.14	0.13	JX017343
Mp07-nfrdi	(AC)_31_	**F** **R**	GTATACAAGTCCCGGTCCTGT CATCCGTGCATGTGTGCCTAT	48	103–173	5	0.675	0.455	JX017344
Mp08-nfrdi	(AC)_37_	**F** **R**	TATAGTTCGGACGGACATGGACA GCCCAAGAGTTGAACATCAGGTT	48	129–227	43	0.603	0.594	JX017345
Mp09-nfrdi	(GT)_5_CT(GT)_7_ CT(GT)_3_	**F** **R**	ACATACGTGTGCGCATGCATGTG GCAGGCGGTTTGGCTGGCAGGTC	58	209–267	6	0.394	0.381	JX017346
Mp10-nfrdi	(AC)_10_	**F** **R**	AGGACCTGATCCTGAACACAC TCTGCATGACTGTCTGTCTCC	48	188	1	-	-	JX017347
Mp11-nfrdi	(GT)_11_	**F** **R**	CCGAGTGCAGAAGAGGAACACAC CACACAGGACCTGATCGTGAACA	48	118	1	-	-	JX017348
Mp12-nfrdi	(AC)_35_	**F** **R**	ACAGACCAAACCATCCTTATCCC CTATTTTCAAGCCAGGCAGAATG	50	93	1	-	-	JX017349
Mp13-nfrdi	(AC)_8_	**F** **R**	TTAACCCGGCCACCCACCTATAC CGTATTTGTGCGCATGCGTGTGC	50	83	1	-	-	JX017350
Mp14-nfrdi	(GT)_10_	**F** **R**	GCAGCAGGCCGAGTGCAGAAG CGTTGGGGCACCGCGACCACA	48	165	1	-	-	JX017351
Mp15-nfrdi	(GT)_11_TT(GT)_16_ (AT)_2_(GT)_2_GA(GT)_9_	**F** **R**	GCCTATATCTGCGTATGTGCATC AGGGGACCGCGATCGTATACAAG	48	112	1	-	-	JX017352

**Table 2:** Variability of alleles at nine microsatellite loci in two populations of *Meretrix petechialis* from Korea.

		Microsatellite loci									
			
Pop.		Mp01-nfrdi	Mp02-nfrdi	Mp03-nfrdi	Mp04-nfrdi	Mp05-nfrdi	Mp06-nfrdi	Mp07-nfrdi	Mp08-nfrdi	Mp09-nfrdi	Means
GC	*N*	35	35	35	34	33	35	35	35	35	34.7
	*N*a	3	5	2	7	2	2	2	18	3	5.3
	*A*_R_	2.8	4.6	2.0	6.5	2.0	2.0	2.0	15.7	2.8	4.9
	*R*	179–199	102–136	149–155	246–286	78–92	168–196	143–173	129–205	209–247	
	*H*o	0.400	0.286	0.114	0.353	0.303	0.057	0.714	0.543	0.571	0.372
	*H*e	0.330	0.260	0.109	0.319	0.261	0.056	0.466	0.549	0.467	0.365
	*FIS*	−0.212	−0.097	−0.045	−0.107	−0.161	−0.015	−0.533 *	0.011	−0.224	−0.084

MA	*N*	58	57	58	58	58	58	58	58	57	57.8
	*N*a	2	6	2	8	2	2	3	26	4	6.7
	*A*_R_	2.0	4.9	2.0	5.5	2.0	2.0	2.5	16.9	3.0	5.2
	*R*	179–199	102–164	149–155	246–280	78–92	168–196	103–173	129–227	209–267	
	*H*o	0.431	0.263	0.138	0.345	0.379	0.155	0.603	0.586	0.333	0.355
	*H*e	0.341	0.243	0.130	0.308	0.331	0.144	0.430	0.651	0.309	0.374
	*FIS*	−0.264	−0.084	−0.065	−0.119	−0.146	−0.075	−0.403 *	0.099	−0.078	−0.051

Mean all pops.	*N*	46.5	46	46.5	46	45.5	46.5	46.5	46.5	46	
	*N*a	2.5	5.5	2	7.5	2	2	2.5	22	3.5	
	*A*_R_	2.4	4.8	2	6	2	1.9914	2.3	16.3	2.9	
	*R*	179–199	102–164	149–155	246–286	78–92	168–196	103–173	129–227	209–267	
	*H*o	0.386	0.275	0.126	0.349	0.341	0.106	0.659	0.565	0.452	
	*H*e	0.336	0.252	0.119	0.314	0.296	0.1	0.448	0.6	0.388	
	*FIS*	−0.238	−0.091	−0.055	−0.113	−0.154	−0.045	−0.468 *	−0.055	−0.151	

Number of samples (*N*); number of alleles per locus (*N*a); allelic richness (*A*_R_); allelic size range (*R*); expected heterozygotity (*H*e); and observed heterozygosity (*H*o) are given for each population and locus.

* Not in conformity with Hardy-Weinberg equilibrium (*p* < 0.01, Bonferroni-corrected value).

**Table 3:** Allele sizes in cross-species amplification of 15 microsatellite loci of *Meretrix petechiails* in 12 related species.

Taxonomy		Veneridae				Psammobiidae	Mactridae			Corbiculidae				

Species	*Meretrix* *petechialis*	*Meretrix* *lousoria*	*Gomphina* *veneriformis*	*Cyclina* *sinensis*	*Ruditapes* *philippinarum*	*Nuttallia* *japonica*	*Mactra* *chinensis*	*Mactra* *veneriformis*	*Tresus* *keenae*	*Corbicula* *japonica*	*Corbicula* *leana*	*Corbicula* *fluminea*	*Corbicula* *colorata*	Transferability *
Number of Individuals	95	7	2	2	9	3	1	2	1	2	2	4	2	
Mp01-nfrdi	179–199	179	-	91–139	-	179–199	-	-	-	-	77	-	83	41.7 (16.7)
Mp02-nfrdi	102–164	-	132	64–124	112–306	222	-	146	146–150	276–334	242–300	82–178	82	83.3 (50.0)
Mp03-nfrdi	149–155	149	-	143	117	-	-	65	-	-	-	-	-	33.3 (0)
Mp04-nfrdi	246–286	-	-	-	-	-	-	-	-	-	-	-	-	0/0
Mp05-nfrdi	78–92	78–92	-	82–332	-	112	-	214	-	108	-	96	-	50.0 (16.7)
Mp06-nfrdi	168–196	168	126–296	126–298	72–122	126–140	196	82–228	164	98	166	-	132	91.7 (33.3)
Mp07-nfrdi	103–173	143–173	-	91–111	-	67	-	-	-	-	155–175	-	93	41.7 (25.0)
Mp08-nfrdi	129–227	-	87–123	89–99	137–245	159–161	169–253	-	215	101	243–245	111–135	245	83.3 (58.3)
Mp09-nfrdi	209–267	-	-	-	-	-	-	-	-	-	-	-	-	0/0
Mp10-nfrdi	188	188	196	192–198	94–108	-	110–178	182	112	266–270	-	100–166	-	75.0 (41.7)
Mp11-nfrdi	118	-	132–138	-	-	132–146	156	-	94–178	-	-	-	138	41.7 (25.0)
Mp12-nfrdi	93	-	65–111	93	95–123	-	-	121–161	-	-	-	-	-	33.3 (25.0)
Mp13-nfrdi	83	-	71	-	65–87	115–215	91–219	91–139	199	-	105	83	-	66.7 (33.3)
Mp14-nfrdi	165	-	157	157	129–131	75	-	101	-	-	-	-	239–243	50.0 (16.7)
Mp15-nfrdi	112	76–112	-	-	102	-	-	222	-	-	-	126–350	-	33.3 (16.7)
Transferability *	-	46.7 (20.0)	53.0 (26.7)	66.7 (40.0)	60 (46.7)	60.0 (33.3)	33.3 (20.0)	60.0 (20.0)	40.0 (13.3)	33.3 (13.3)	40.0 (20.0)	40.0 (26.7)	46.7 (6.7)	

* Numbers in parentheses represent the percentage of polymorphic loci.
